# Pathogenic *E. coli* Exploits SslE Mucinase Activity to Translocate through the Mucosal Barrier and Get Access to Host Cells

**DOI:** 10.1371/journal.pone.0117486

**Published:** 2015-03-19

**Authors:** Maria Valeri, Silvia Rossi Paccani, Magdalena Kasendra, Barbara Nesta, Laura Serino, Mariagrazia Pizza, Marco Soriani

**Affiliations:** 1 Novartis Vaccines and Diagnostics S.r.l., Via Fiorentina 1, Siena, Italy; 2 Children’s Hospital Boston, Harvard Medical School, 200 Longwood Avenue, Boston, Massachusetts 02115, United States of America; Indian Institute of Science, INDIA

## Abstract

SslE is a zinc-metalloprotease involved in the degradation of mucin substrates and recently proposed as a potential vaccine candidate against pathogenic *E. coli*. In this paper, by exploiting a human *in vitro* model of mucus-secreting cells, we demonstrated that bacteria expressing SslE have a metabolic benefit which results in an increased growth rate postulating the importance of this antigen in enhancing *E. coli* fitness. We also observed that SslE expression facilitates *E. coli* penetration of the mucus favouring bacteria adhesion to host cells. Moreover, we found that SslE-mediated opening of the mucosae contributed to the activation of pro-inflammatory events. Indeed, intestinal cells infected with SslE-secreting bacteria showed an increased production of IL-8 contributing to neutrophil recruitment. The results presented in this paper conclusively designate SslE as an important colonization factor favouring *E. coli* access to both metabolic substrates and target cells.

## Introduction

*E. coli* is mainly regarded as a commensal microorganism retaining probiotic property [1]. However, some bacteria possess virulence factors that allow them to cause intestinal and extra-intestinal diseases [2]. Pathogenic *E. coli* species principally reside in the gut, but could also migrate to distal organs such as bladder and kidney, where they can cause urinary tract infections and sepsis. *E. coli* pathogenesis is characterized by IL-8 secretion and a strong infiltration of polymorphonuclear leukocytes [3–6]. In order to colonize or invade intestinal epithelium, *E. coli* must penetrate the mucus barrier and then either attach to the apical surface of epithelial cells or release toxins that disrupt epithelial integrity [7]. The mucus layer, largely composed of mucins, contains various digestive enzymes and antimicrobial peptides as well as immunoglobulins. The inner layer is densely packed, firmly attached to the epithelium, and devoid of bacteria. In contrast, the outer layer is movable and has an expanded volume that favours bacterial colonization [8,9]. Notably, bacterial pathogens have evolved mechanisms to circumvent this mucus hurdle and directly access the epithelial surface [10,11].

The recent description of SslE as a novel *E. coli* mucinase [12,13], has opened new outlooks on the mechanisms used by this important mucosal pathogen to adapt to the intestine. SslE (ECOK1_3385) is a promising vaccine candidate identified by using a subtractive reverse vaccinology approach [14].The antigen is characterized by the presence of a M60-like domain representative of a new extracellular zinc-metalloprotease sub-family which is implicated in glycan recognition and processing. SslE is a 160 kDa mucin-binding protein able to degrade intestinal mucins including Muc2, Muc3 and bovine submaxillary mucin [12,13]. However, the contribution of this protein to *E. coli* adaptation to the host still remains controversial. Indeed, SslE also appears to be required for biofilm formation in an EPEC strain [15], although this was not confirmed in an atypical EPEC strain [16]. Thus, the function of SslE remains to be fully elucidated.

In the present study, we show that SslE expression not only increases bacterial growth in the presence of mucosal substrates but it also facilitates *E. coli* penetration of the mucus. The evidence that SslE expressing bacteria have an enhanced access to the apical epithelial surface was corroborated by an increased pro-inflammatory response. These results further support the pivotal role of SslE during *E. coli* colonization of the intestinal mucosa.

## Materials and Methods

### Antibodies, reagents and recombinant proteins

Antibody against muc-5AC and muc-3 were from Sigma-Aldrich (Milan, Italy), Anti-muc2 and muc3 antibodies were from Abcam, anti-muc1 was from Thermo Fisher Scientic, Alexa Fluor 568 anti-mouse secondary antibody and ProLong Gold Antifade Reagent with DAPI were obtained from Invitrogen.

Cells were maintained Dulbecco’s Modified Eagle Medium (DMEM) or in Roswell Park Memorial Institute medium (RPMI), supplemented with 10% heat-inactivated fetal bovine serum, non-essential amino acids and 2 mM L-glutamax (Invitrogen Ltd, Paisley, UK). Blood neutophils were isolated by stratifying whole blood on Ficoll-Paque Plus (GE Healthcare).

For cDNA preparation we used Directzol RNA kit (Zymo Research) and TURBO DNase (Life Technologies), the real time analyses were performed in PCR plates using FastStart Universal SYBR Green Master (Roche Diagnostics).

### Ethics statement

The institutional review board of the Department of Health Service at Novartis Vaccines and Diagnostics (Siena, Italy) approved the study and the use of human samples from the volunteers. Written, informed consent was obtained from the healthy donors (available from authorized blood banks).

### Bacterial strains and culture conditions

ExPEC IHE3034 (serotype O18 K1:H7), was isolated in Finland in 1976 from a case of human neonatal meningitis [17]. Strains were cultured in Luria-Bertani broth at 37°C with agitation and aeration. *SslE* deletion mutant and complemented strains have been previously described [12]. Bacterial growth was performed by sub-culturing overnight broth cultures into the appropriate medium and reading the optical density at 600 nm (OD_600_) at various time points. Growth in minimal medium was performed in M9 medium with 1% glucose; 0.05% glucose was employed during experiments in which mucin was added. Mucus was pooled from confluent HT29-MTX at 13,000g for 30 min at 4°C [11]. The clones carrying a specific antibiotic resistance cassette were grown in the presence of kanamycin (50 μg/ml) or erythromycin (50 μg/ml).

### Cell culture

HT29-MTX human intestinal epithelial cells [18] derived from a colonic carcinoma were progressively adapted to a galactose-containing media [19], for experiments cells were grown as 2-dimensional (2D) monolayers on collagen coated Transwell inserts (0.4-μm pore size; BD Biosciences) and, unless stated otherwise, allowed to differentiate for 21 days.

Polymorphonuclear neutrophils were purified from buffy coats by density gradient centrifugation (400 × g for 30 min at room temperature) on Ficoll-Paque Plus, followed by centrifugation (250 × g for 10 min at 4°C) on a 3% (wt/vol) dextran solution. After osmotic lysis of erythrocytes, cells were resuspended in RPMI 1640 supplemented with 10% FBS and incubated at 37°C in a humidified atmosphere with 5% CO2

### Measurement of trans-epithelial electrical resistance (TEER)

The integrity of polarized HT29-MTX monolayers was checked by measurement of Trans Epithelial Electrical Resistance (TEER) using an EVOMAX meter and STX-2 probe (World Precision Instruments). TEER was measured at different time points over a 21 day culture period and expressed as Ω/cm^2^.

### RNA isolation and RT-PCR

Total RNA was isolated both from HT29-MTX cells and from bacteria using Directzol RNA kit and was treated with DNase. The RNA was ethanol precipitated and dissolved in 30 μl RNase-free water. Real-time quantitative PCR was performed in a LightCycler 480 II real-time PCR system (Roche Diagnostics). All samples were run in triplicate on 96-well optical PCR plates. The specific primers used to amplify cDNA fragments are listed in Table 1. After an initial denaturation at 95°C for 10 min, denaturation in the subsequent 40 cycles was performed at 95°C for 15 s, followed by primer annealing at 60°C for 30 s and a final extension at 72°C for 30 s. For relative quantification of gene expression, the starting mRNA copy number of the unknown samples was determined using the comparative threshold cycle (ΔΔC_***T***_) method, as previously described [20], and levels of the different transcripts were normalized to 16S rRNA or β-actin, used as housekeeping genes.

**Table 1:** Primers pair used in this study.

Gene	Primers Pair	Reference
SslE	F-CTCATCTTCCTTGCCCTCTTC	This study
	R- TCATGGAGTCGAGTTGCAGA	This study
16S	F- ACGTGCTACAATGGCGCATA	Ref-[34]
	R- TCATGGAGTCGAGTTGCAGA	Ref-[34]
Muc1	F-TCAGCTTCTACTCTGGTGCACAA	Ref-[35]
	R-ATTGAGAATGGAGTGCTCTTGCT	Ref-[35]
Muc2	F-CTGCACCAAGACCGTCCTCATG	Ref-[36]
	R-GCAAGGACTGAACAAAGACTCAGAC	Ref-[36]
Muc3	F-AGTCCACGTTGACCACTGC	Ref-[37]
	R-TGTTCACATCCTGGCTGGCG	Ref-[37]
Muc4	F-CGCGGTGGTGGAGGCGTTCTT	Ref-[36]
	R-GAAGAATCCTGACAGCCTTCA	Ref-[36]
Muc5	F-TGATCATCCAGCAGCAGGGCT	Ref-[36]
	R-CCGAGCTCAGAGGACATATGGG	Ref-[36]
ACTIN	F-GCTATCCCTGTACGCCTCTG	Ref-[38]
	R-CTCCTTCTGCATCCTGTCGG	Ref-[38]

### Bacterial growth in HT29-MTX intestinal epithelial cell mucus and infection assay

Three set of transwells of polarized HT29-MTX cells were infected with *E. coli* at a multiplicity of infection (MOI) of 100. After 2 hrs of incubation wells were washed and a total association assay was performed on one set of wells. The remaining two sets of wells were further incubated at air-liquid interface for 24 or 48 h, and then CFU counting was performed. The same experiment was run on non-differentiated HT29-MTX cells (non-polarized and no mucus), as control. For the infection assays polarized HT29-MTX cells were grown on transwell filters as described above. Infections were performed in triplicate in DMEM without serum at a MOI of 100 bacteria per cell at 37°C, 10% CO2, for 4 hrs. Then wells were washed and 10 mM N-acetylcysteine (PBS 0.2 mM calcium chloride, 0.5 mM magnesium chloride and 15 mM glucose) was added for 1 h with agitation at 70 rpm, to remove the mucus layer after the *E. coli* infection period [21]. To evaluate the bacteria trapped into mucus, serial dilutions of the NAC medium were plated. For the quantitative determination of the cell-associated *E. coli*, infected cells were lysed with 1% saponin for 10 min, and serial dilutions of the cell lysates were made.

### Enzyme-linked immunosorbent assay (ELISA)

Post *E. coli* infection, HT-29-MTX cell supernatant was collected and analyzed for IL-8 using a Human IL-8 ELISA Kit (R&D Systems, QuantakineR) according to the manufacturer’s directions. The intra-assay coefficient of variation (CV) and the inter-assay CV were <5%.

### Chemotaxis assay

To measure neutrophil chemotaxis, bottom chambers of transwell supports were filled with supernatants deriving from HT29-MTX cells infected for 4 hours with IHE3034 or IHE3034ΔsslE strains. Neutrophils (2.5 × 10^5^) were added to the upper chambers. After 1h at 37°C, cells that had migrated toward the lower compartments were quantified by flow cytometry. Cells were analyzed with a LSRII flow cytometer (Beckton-Dickinson) by using Floujo software.

### Statistical Analysis

Mean values, standard deviation values and non-parametric Mann-Whitney U test were calculated using the GraphPad Prism 6 application. A level of P<0.05 was considered statistically significant.

## Results

### SslE expression is modulated by contact with differentiated mucus producing cells

**Fig 1:**
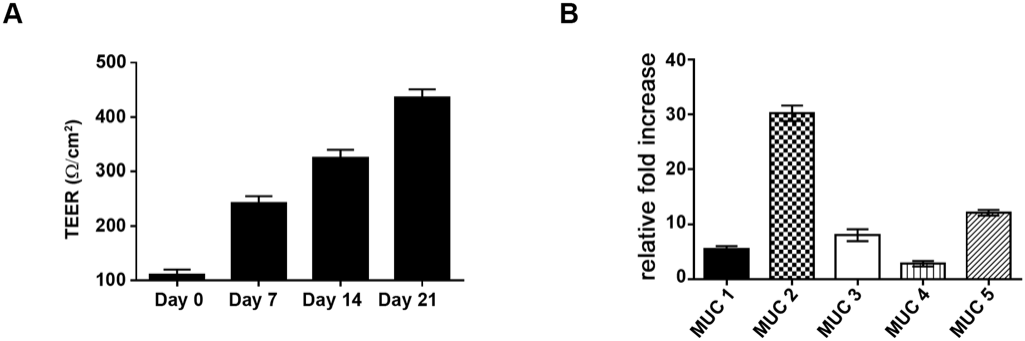
Kinetics of transepithelial electrical resistance in HT29-MTX cells over a 21 day period of differentiation. (A) TEER values were measured at different time points throughout a 21 day period of differentiation. (B) RT-PCR analysis of MUC mRNAs. After 21 days of differentiation, HT29-MTX mRNA was isolated, and cDNA was used to compare the level of MUC gene expression. Data are represented as relative fold increase of MUC mRNA in differentiated mucus-producing cells *versus* non-differentiated (control) cells. Control cells were assigned a value of 1.0. Levels of the different transcripts were normalized to β-actin, used as a house keeping gene. Error bars represent the SD.

**Fig 2:**
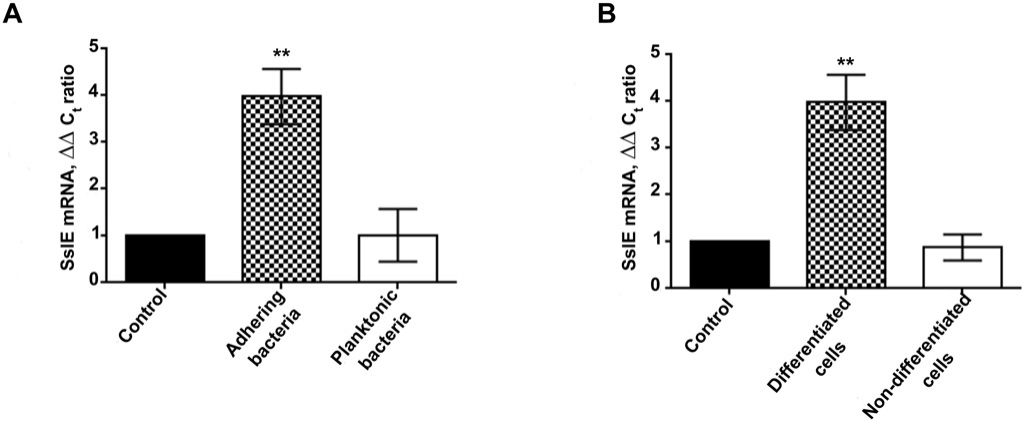
Modulation of SslE gene expression upon interaction with intestinal epithelial cells. (A) SslE transcription level in bacteria adhering to differentiated cells and in planktonic organisms. (B) SslE transcription level upon the interaction with differentiated or not differentiated HT29-MTX cells. Starting mRNA copy number of the unknown samples was determined using the comparative ΔΔ_***CT***_ method, and levels of the different transcripts were normalized to 16S rRNA, used as a housekeeping gene. Error bars represent the SD. **, P ≤ 0.01. n = 3.

To dissect the contribution of SslE to *E. coli* infection of mucosal surfaces, we used an *in vitro* model based on polarized and fully differentiated HT29-MTX human colonic epithelial cells [18]. The degree of epithelial polarity was monitored by measuring trans-epithelial electrical resistance (TEER) (Fig. 1A), while the expression of both secreted and cell-surface mucins was assessed by qRT-PCR and confocal microscopy analysis (Fig. 1B and S1 Fig.). To investigate whether the interaction with intestinal epithelial cells modulates SslE expression, we examined the transcription profile of bacteria adhering to differentiated HT29-MTX cells. To this end polarized cells were infected for 30 min and then SslE expression in bacteria adhering to mucus-producing cells was compared with that of bacteria growing in medium alone. As reported in Fig. 2A, a statistically significant increase in *sslE* transcript was observed during the interaction of *E. coli* with epithelial cells. Western blot analysis of supernatants from bacteria incubated in medium alone or HT29-MTX cells confirmed an increased production of SslE in the presence of differentiated cells (S2 Fig.). To further understand whether bacteria-cell contact or factors released in the medium were responsible for gene activation, the level of SslE transcription of cell-adhering bacteria was compared with that of planktonic bacteria collected from the supernatant. Under such conditions, we clearly demonstrated that SslE expression is significantly increased only in cell-adhering bacteria (Fig. 2A) suggesting that host cells surface components are required to trigger the activation of the gene. Ultimately, the contribution of cell glycocalyx to the protein up-regulation was evaluated by comparing the level of SslE expression upon the interaction with differentiated mucus-producing cells *versus* non-differentiated ones. Interestingly, transcription of the *sslE* gene was not affected by the contact with the non-differentiated cells (Fig. 2B). Collectively, these data postulate that host cell differentiation status is crucial for the modulation of SslE expression.

### SslE mucinase activity facilitates *E. coli* colonization of the apical cell surface by improving bacterial growth rate

**Fig 3:**
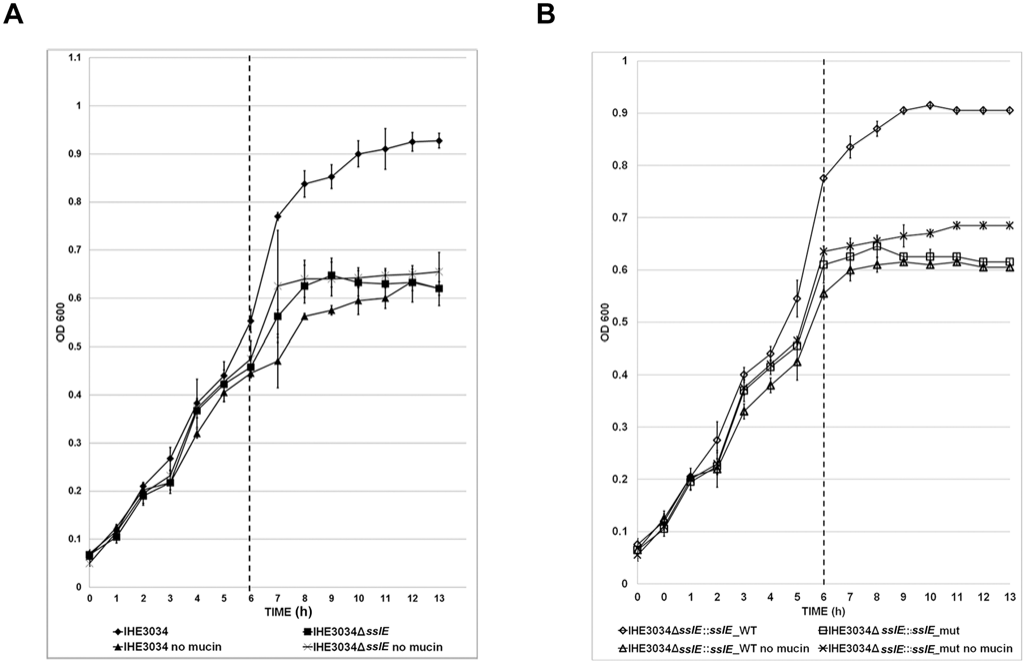
Growth curves of strain IHE3034 in the presence of mucin. Growth curves of (A) IHE3034 and IHE3034Δ*sslE* strains (B) IHE3034Δ*sslE*::*sslE*_WT and IHE3034Δ*sslE*::*sslE*_mut, are shown in M9 minimal medium with and without the addition of mucin harvested from HT29-MTX cells after 6 h of incubation (Dash line). Measurements were performed in triplicate.

**Fig 4:**
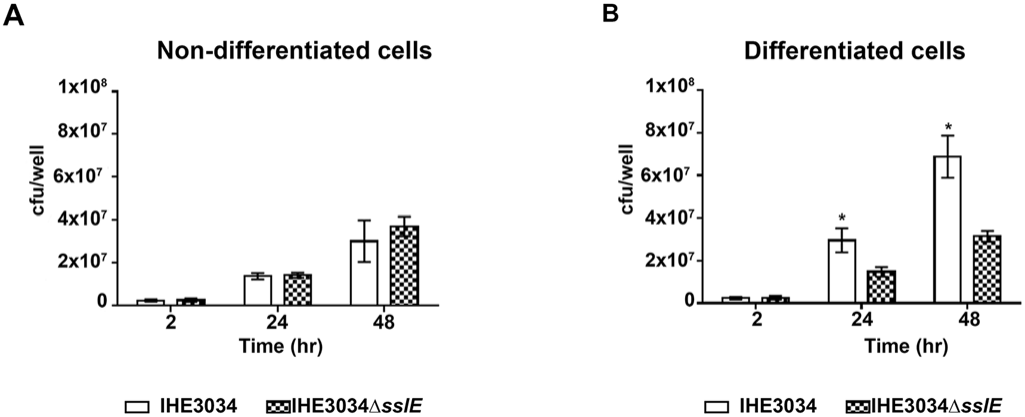
*E. coli* growth rate in association with HT29-MTX cells. Using the transwell system, three sets of non-differentiated cells (A) and three sets of differentiated-mucus—producing- cells (B) were infected with WT and *sslE* KO bacteria for 2 hrs. Medium was removed to eliminate non-adhering bacteria and two sets of wells were used to do a total association assay, while the other wells were further incubated for 24 and 48 hrs at air liquid interface. At the end of the incubation period a total association assay was performed. The data presented are means ± standard deviations for 3 replicate experiments (n = 9).*P≤0.05. Error bars, SD.

To assess whether SslE expression could increase the overall fitness of *E. coli*, we performed growth curves of IHE3034 wild-type and IHE3034*ΔsslE* knock-out mutant strains in M9 minimal medium containing mucus harvested from HT29-MTX cells. As shown in Fig. 3A, the presence of mucins boosted the growth ability of the wild type strain which reached a plateau at OD_600_ = 0.9 (stationary phase), whereas growth rate of the *sslE* mutant strain was unaffected. Similar results were obtained using the IHE3034Δ*sslE*::*sslE*_WT complemented strain carrying a WT *sslE* gene and HE3034Δ*sslE*::*sslE*_mut carrying a triple mutation in the metallopeptidase motif (**YV**VG**Y** vs. HEVGH) [12] (Fig. 3B). To demonstrate that SslE-mediated mucin degradation by increasing bacterial growth rate facilitates colonization of apical surfaces, we compared IHE3034 wild-type and IHE3034*ΔsslE* mutant strains for the ability to reside on mucus producing cells. As shown in Fig. 4, infection with WT and *ΔsslE* strains for 2 hours resulted in almost an equal bacteria binding to both non-differentiated and differentiated HT29-MTX cells (Fig. 4A and 4B, respectively). However, prolonged incubations at air liquid interface for up to 24 and 48 hours, revealed statistically significant differences in the number of WT bacteria growing on mucus producing cells compared to the *sslE* mutant (Fig. 4B). Such differences were abrogated when WT and *ΔsslE* infections were performed using non-differentiated cells (Fig. 4A). These data suggest that SslE expressing bacteria may increase their fitness by using mucosal apical glycoproteins (including mucins).

### SslE contributes to *E. coli* translocation of the mucosal barrier *in vitro*

**Fig 5:**
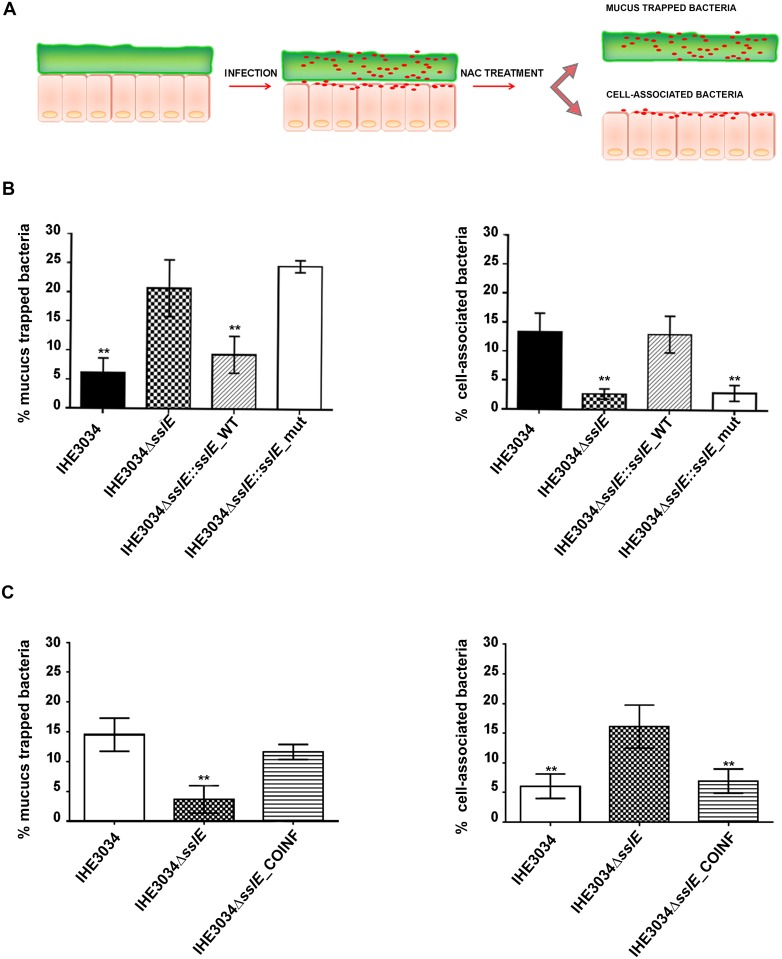
SslE contributes to the capacity of IHE3034 strain to reach the surface of mucus-producing epithelial cells. (A) Schematic representation of the experimental procedure. (B), Percentage of mucus trapped (left panel) and cell associated (right panel) **I**HE3034 (WT), IHE3034Δ*sslE* (KO), IHE3034Δ*sslE*::*sslE*_WT (COMPL) and IHE3034Δ*sslE*::*sslE*_mut (MUT) bacteria after 4hrs of infection. *n* = 4, **P≤0.01; Error bars, SD; percentages were calculated on recovered CFU respect to the starting inoculum. (C) Co-infection experiments: percentage of mucus trapped (left panel) and cell associated (right panel) **I**HE3034 (WT), IHE3034Δ*sslE* (KO) or IHE3034Δ*sslE* plus IHE3034 (KO_COINF) bacteria. Strains were plated on both non-selective and selective plates to differentiate WT and Δ*sslE* for CFU counts. *n* = 4, **P≤0.01; Error bars, SD;

In order to further reinforced our previous finding on the mucolytic activity of the protein, observed using gel matrix [12], and to evaluate its role in host colonization process, we tested SslE activity in our *in vitro* gut model. Polarized monolayers of mucus producing cell were infected for 4 hours with the wild-type IHE3034, its isogenic derivative IHE3034Δ*sslE*, IHE3034Δ*sslE*::*sslE*_WT and HE3034Δ*sslE*::*sslE*_mut. Addition of N-acetyl cysteine (NAC) at the end of the incubation period allowed us to remove the apical mucus layer and to distinguish between the bacteria trapped in it and the bacteria adhering to underlying cells (Fig. 5A). As shown in Fig. 5B, the *sslE* deficient strain was less efficient in reaching the cell surface compared to the isogenic WT. Indeed, a high number of IHE3034Δ*sslE* and IHE3034Δ*sslE*::*sslE*_mut bacteria were recovered from the mucus fraction, while both WT and the complemented strains were mainly associated to the cells underlying the mucus layer. These data confirm that SslE facilitates *E. coli* penetration of mucus and allows bacteria to get access to the host cells surface. Of interest, co-infection experiments using IHE3034 WT and IHE3034Δ*sslE* strains revealed that the expression of SslE by the WT strain complements the ability of the *sslE* deficient strain to get access to the apical cell surface of the HT29-MTX polarized epithelium (Fig. 5C).

### SslE-mediated opening of the mucosae contributes to the activation of pro-inflammatory events

**Fig 6:**
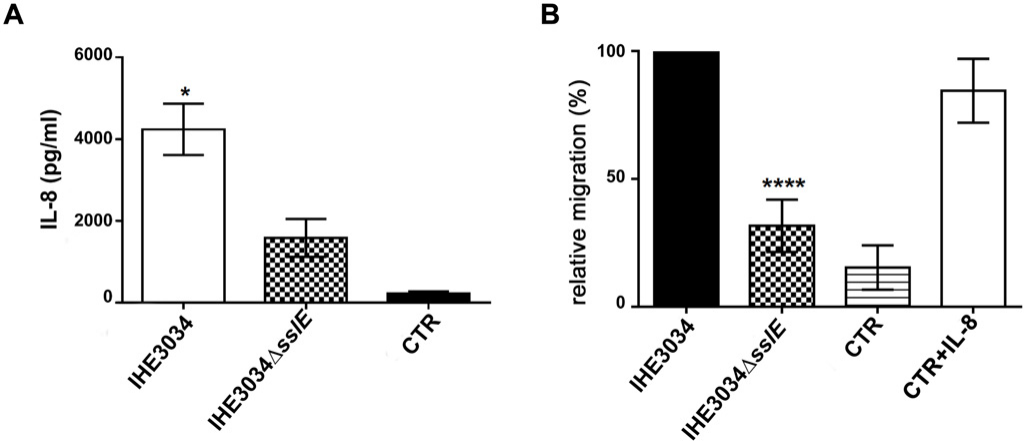
SslE induces IL-8 secretion and stimulates neutrophil chemotaxis. (A) IL-8 levels in supernatants from the apical compartments of polarized HT29-MTX cells infected with IHE3034 WT and IHE3034Δ*SslE* KO bacteria. Data show mean chemokine concentrations in culture supernatants representative of three independent experiments *P≤0.05. Error bars, SD. (B) To measure neutrophil chemotaxis, bottom chambers of transwell supports were filled with supernatants deriving from HT29-MTX infected cells. Neutrophils were added to the upper chambers. After 1 h at 37°C, cells that had migrated toward the lower compartments were quantified by flow cytometry. DMEM has been used as a negative control and recombinant IL-8 as a positive control. The graph represents a typical experiment out of three performed with similar results. **** P ≤ 0.0001. Error bars, SD.

As a result of the interaction with pathogens, the enterocytes act as immunocompetent cells and secrete various signalling molecules, such as cytokines and chemokines. Previous work from Svanborg’s group [6] has clearly shown that *E. coli* species targeting epithelial cells induce soluble mediators leading to the recruitment of inflammatory cells that participate directly in the clearance of bacteria. To further prove that SslE expression favours *E. coli* access to epithelial cells, we investigated whether this phenotype correlates with an increased pro-inflammatory response. To this end, supernatants derived from HT29-MTX cells infected with the wild-type IHE3034 or its isogenic Δ*sslE* mutant strains were collected. We observed that both bacterial strains were able to stimulate IL-8 release compared to negative control (supernatant of uninfected cell). Of note, WT strain induced a statistically significant higher level of IL-8 compared to the Δ*sslE* strain (Fig. 6A); further supporting our hypothesis that SslE is indirectly involved in promoting inflammation. Since IL-8 is a well-known chemo-attractant able to stimulate neutrophil recruitment [6], we compared supernatants derived from cells infected with WT and Δ*sslE* bacteria for their ability to promote neutrophil migration. As expected, flow cytometric analysis revealed that WT-derived supernatant induced a higher PMN migration compared to KO (Fig. 6B).

## Discussion

Multiple mucus layers overlying gut epithelium act as microbial sensing and intrinsic defence systems that counteract against infective intruders. They protect from hurdle colonization, invasion, and systemic dissemination of both symbiotic and pathogenic microorganisms [22]. An important aspect in the understanding of microbial pathogenesis is the recognition of the different strategies evolved by microbes to circumvent the mucosal barrier and cause disease [23]. A number of virulence factors such as proteases, glycosidases, and mucin secretagogues are produced by these organisms and are believed to be responsible for disruption and depletion of the mucus gel [24,25]. Serine protease such as Pic [7], zinc metalloproteases (StcE, Hap) [26,27], as well as mucin-degrading enzymes [28], metabolize mucin oligosaccharides, reduce mucus viscosity and release antimicrobial peptides. The recent identification of SslE, a novel *E. coli* mucinase [12,13], has opened new outlooks on the way this mucosal pathogen adapts to the inhospitable environment of human intestine. To elucidate the contribution of SslE to *E. coli* pathogenesis, we developed an *in vitro* model based on colonic HT29-MTX cells grown on transwell inserts. This system resembles the intestinal mucosa with the presence of both gel forming and secreted mucins. In particular, we demonstrated that SslE facilitates penetration of the mucus layer and the consequent access to the underlying epithelial cells. These results further reinforce the hypothesis recently formulated by our group (confirmed also by Fleckenstein and colleagues [12,13]) for a role of SslE in the colonization of the intestine. Notably, the use of the N-acetyl cysteine allowed us to precisely distinguish between bacteria trapped into the mucus network and bacteria bound to underlying cells. Our results clearly establish a cause-effect relationship between SslE expression and mucus barrier penetration. In particular, we show that bacteria with an impaired expression of SslE get trapped in the sticky mucosal matrix witch restrict their ability to spread, leading them to a reduced infectiveness. Of importance, our evidence that SslE expression is augmented when bacteria are in contact with the differentiated mucus-producing cells not only postulates the specificity of SslE for mucosal surfaces, but also its active role during colonization process.

The ability of indigenous microflora to multiply at a rate that allows counteracting the turnover and the erosion of the mucus layer is crucial for their capacity to persist in an overcrowded niche such as the human gut. In this context, mucins represent preferential substrates for several human colonizers including avirulent *Escherichia coli* [29], *Salmonella typhimurium* [30], *Clostridium perfringens* [31], *Bacteroides* species [32] and virulent *Shigella flexneri* [33]. The fact that SslE expression, by mediating mucins catabolism, not only favours *E. coli* diffusion but also enhances its growth rate, suggest a broad impact of this protein on the overall bacterial fitness. Of importance, this was demonstrated in the presence of mucins extracted from cells as well as when bacteria were in direct contact with the mucus film lying on top of differentiated HT29-MTX cells. However, whether the advantage of SslE results from the release of glycans from intestinal mucus and/or from degradation of the mucin protein backbone remain to be elucidated.

**Fig 7:**
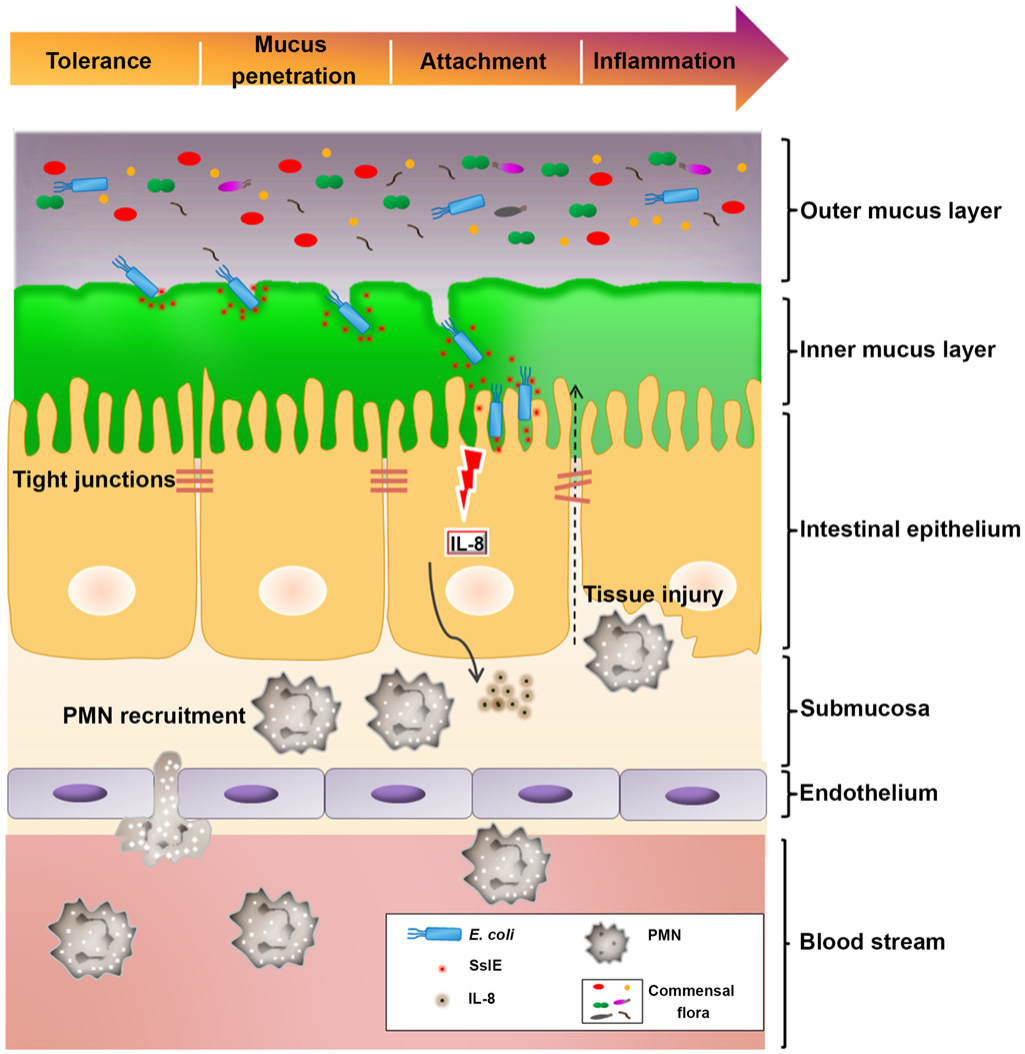
Schematic representation of the contribution of SslE to *E.coli* pathogenesis. Gut mucus forms two layers, an inner firm mucus layer devoid of bacteria, and an outer layer that is not sterile and is a major habitat for commensal bacteria. *E. coli* can penetrate this barrier through the SslE-mediated enzymatic degradation of the mucus, targeting epithelial cells. This interaction will eventually lead to IL-8 release and neutrophils recruitment.

In view of our findings, we propose a model for SslE involvement in *E. coli* translocation of the mucus layer and the consequent triggering of a pro-inflammatory immune response. Briefly, *E. coli* microorganisms, that reside in the outer mucus layer, by their intrinsic mucinase activity may access to the inner mucus barrier and migrate towards cellular targets (Fig. 7). The direct contact of bacteria with the intestinal epithelium leads to the secretion of chemokines and stimulates neutrophil transmigration. The fact that SslE appears to be also secreted by commensal *E. coli* species raises an important issue on the role of this antigen in the adaption to the host. Indeed, commensals are rarely found in the inner mucus barrier of the gut suggesting that *in vivo* SslE activity alone may not be sufficient to allow mucus penetration, but may require the synergy with other proteases expressed by pathogenic strains. We are far from understanding this aspect and more studies are needed to unravel the different strategies used by this pathogen to get access to host cell targets.

In conclusion, by providing new clues on the role of SslE in the interplay between *E. coli* and host cells, we will not only increase the current understanding on *E. coli* pathogenesis, but also better define the mechanism at the base of the protective response induced by this promising vaccine candidate.

## Supporting Information

S1 Fig Characterization of the extracellular mucus layer of HT29-MTXcells. HT29-MTX cells grown on transwell filters for 21 days were stained with specific antibodies for MUC1, MUC2, MUC3, MUC4,MUC5AC and PAN anti-gastric mucin. The mucins are stained in green and the actin skeleton in red. DAPI (blue) staining was used to visualize cell nuclei.
(TIF)

S2 Fig SslE production is enhanced upon contact with epithelial cells. Immunoblot analysis of SslE expression in S/N of bacteria incubated with medium alone (control) or differentiated HT29-MTX cells. A representative experiment out of 3 performed is shown. Molecular weight markers are indicated on the left column.
(TIF)

## References

[1] Deriu E, Liu JZ, Pezeshki M, Edwards RA, Ochoa RJ, et al (2013) Probiotic bacteria reduce salmonella typhimurium intestinal colonization by competing for iron. Cell Host Microbe 14: 26–37. 10.1016/j.chom.2013.06.007 23870311 PMC3752295

[2] Kaper JB (2005) Pathogenic Escherichia coli. Int J Med Microbiol 295: 355–356. 16238012 10.1016/j.ijmm.2005.06.008

[3] Uehling DT, Johnson DB, Hopkins WJ (1999) The urinary tract response to entry of pathogens. World J Urol 17: 351–358. 10654365 10.1007/s003450050160

[4] McCormick BA, Colgan SP, Delp-Archer C, Miller SI, Madara JL (1993) Salmonella typhimurium attachment to human intestinal epithelial monolayers: transcellular signalling to subepithelial neutrophils. J Cell Biol 123: 895–907. 8227148 10.1083/jcb.123.4.895 PMC2200157

[5] Savkovic SD, Koutsouris A, Hecht G (1996) Attachment of a noninvasive enteric pathogen, enteropathogenic Escherichia coli, to cultured human intestinal epithelial monolayers induces transmigration of neutrophils. Infect Immun 64: 4480–4487. 8890195 10.1128/iai.64.11.4480-4487.1996 PMC174401

[6] Svanborg C, Godaly G, Hedlund M (1999) Cytokine responses during mucosal infections: role in disease pathogenesis and host defence. Curr Opin Microbiol 2: 99–105. 10047563 10.1016/s1369-5274(99)80017-4

[7] Henderson IR, Czeczulin J, Eslava C, Noriega F, Nataro JP (1999) Characterization of pic, a secreted protease of Shigella flexneri and enteroaggregative Escherichia coli. Infect Immun 67: 5587–5596. 10531204 10.1128/iai.67.11.5587-5596.1999 PMC96930

[8] McGuckin MA, Linden SK, Sutton P, Florin TH (2011) Mucin dynamics and enteric pathogens. Nat Rev Microbiol 9: 265–278. 10.1038/nrmicro2538 21407243

[9] Dharmani P, Srivastava V, Kissoon-Singh V, Chadee K (2009) Role of intestinal mucins in innate host defense mechanisms against pathogens. J Innate Immun 1: 123–135. 10.1159/000163037 20375571 PMC7312850

[10] Kim M, Ashida H, Ogawa M, Yoshikawa Y, Mimuro H, et al (2010) Bacterial interactions with the host epithelium. Cell Host Microbe 8: 20–35. 10.1016/j.chom.2010.06.006 20638639

[11] Sperandio B, Fischer N, Joncquel Chevalier-Curt M, Rossez Y, Roux P, et al (2013) Virulent Shigella flexneri affects secretion, expression, and glycosylation of gel-forming mucins in mucus-producing cells. Infect Immun 81: 3632–3643. 10.1128/IAI.00551-13 23876800 PMC3811771

[12] Nesta B, Valeri M, Spagnuolo A, Rosini R, Mora M, et al (2014) SslE elicits functional antibodies that impair in vitro mucinase activity and in vivo colonization by both intestinal and extraintestinal Escherichia coli strains. PLoS Pathog 10: e1004124 10.1371/journal.ppat.1004124 24809621 PMC4014459

[13] Luo Q, Kumar P, Vickers TJ, Sheikh A, Lewis WG, et al (2014) Enterotoxigenic Escherichia coli secretes a highly conserved mucin-degrading metalloprotease to effectively engage intestinal epithelial cells. Infect Immun 82: 509–521. 10.1128/IAI.01106-13 24478067 PMC3911403

[14] Moriel DG, Bertoldi I, Spagnuolo A, Marchi S, Rosini R, et al (2010) Identification of protective and broadly conserved vaccine antigens from the genome of extraintestinal pathogenic Escherichia coli. Proc Natl Acad Sci U S A 107: 9072–9077. 10.1073/pnas.0915077107 20439758 PMC2889118

[15] Baldi DL, Higginson EE, Hocking DM, Praszkier J, Cavaliere R, et al (2012) The type II secretion system and its ubiquitous lipoprotein substrate, SslE, are required for biofilm formation and virulence of enteropathogenic Escherichia coli. Infect Immun 80: 2042–2052. 10.1128/IAI.06160-11 22451516 PMC3370571

[16] Hernandes RT, De la Cruz MA, Yamamoto D, Giron JA, Gomes TA (2013) Dissection of the role of pili and type 2 and 3 secretion systems in adherence and biofilm formation of an atypical enteropathogenic Escherichia coli strain. Infect Immun 81: 3793–3802. 10.1128/IAI.00620-13 23897608 PMC3811761

[17] Achtman M, Mercer A, Kusecek B, Pohl A, Heuzenroeder M, et al (1983) Six widespread bacterial clones among Escherichia coli K1 isolates. Infect Immun 39: 315–335. 6218094 10.1128/iai.39.1.315-335.1983 PMC347943

[18] Lesuffleur T, Barbat A, Dussaulx E, Zweibaum A (1990) Growth adaptation to methotrexate of HT-29 human colon carcinoma cells is associated with their ability to differentiate into columnar absorptive and mucus-secreting cells. Cancer Res 50: 6334–6343. 2205381

[19] Huet C, Sahuquillo-Merino C, Coudrier E, Louvard D (1987) Absorptive and mucus-secreting subclones isolated from a multipotent intestinal cell line (HT-29) provide new models for cell polarity and terminal differentiation. J Cell Biol 105: 345–357. 3611191 10.1083/jcb.105.1.345 PMC2114933

[20] Livak KJ, Schmittgen TD (2001) Analysis of relative gene expression data using real-time quantitative PCR and the 2(-Delta Delta C(T)) Method. Methods 25: 402–408. 11846609 10.1006/meth.2001.1262

[21] Alemka A, Clyne M, Shanahan F, Tompkins T, Corcionivoschi N, et al (2010) Probiotic colonization of the adherent mucus layer of HT29MTXE12 cells attenuates Campylobacter jejuni virulence properties. Infect Immun 78: 2812–2822. 10.1128/IAI.01249-09 20308300 PMC2876579

[22] Madara JL, Nash S, Moore R, Atisook K (1990) Structure and function of the intestinal epithelial barrier in health and disease. Monogr Pathol: 306–324. 2406578

[23] Sansonetti PJ (2004) War and peace at mucosal surfaces. Nat Rev Immunol 4: 953–964. 15573130 10.1038/nri1499

[24] Connaris S, Greenwell P (1997) Glycosidases in mucin-dwelling protozoans. Glycoconj J 14: 879–882. 9511996 10.1023/a:1018554408558

[25] Schneider DR, Parker CD (1982) Purification and characterization of the mucinase of Vibrio cholerae. J Infect Dis 145: 474–482. 7069228 10.1093/infdis/145.4.474

[26] Silva AJ, Pham K, Benitez JA (2003) Haemagglutinin/protease expression and mucin gel penetration in El Tor biotype Vibrio cholerae. Microbiology 149: 1883–1891. 12855739 10.1099/mic.0.26086-0

[27] Grys TE, Siegel MB, Lathem WW, Welch RA (2005) The StcE protease contributes to intimate adherence of enterohemorrhagic Escherichia coli O157:H7 to host cells. Infect Immun 73: 1295–1303. 15731026 10.1128/IAI.73.3.1295-1303.2005 PMC1064933

[28] Mantle M, Rombough C (1993) Growth in and breakdown of purified rabbit small intestinal mucin by Yersinia enterocolitica. Infect Immun 61: 4131–4138. 8406802 10.1128/iai.61.10.4131-4138.1993 PMC281135

[29] Wadolkowski EA, Laux DC, Cohen PS (1988) Colonization of the streptomycin-treated mouse large intestine by a human fecal Escherichia coli strain: role of growth in mucus. Infect Immun 56: 1030–1035. 3281898 10.1128/iai.56.5.1030-1035.1988 PMC259757

[30] McCormick BA, Stocker BA, Laux DC, Cohen PS (1988) Roles of motility, chemotaxis, and penetration through and growth in intestinal mucus in the ability of an avirulent strain of Salmonella typhimurium to colonize the large intestine of streptomycin-treated mice. Infect Immun 56: 2209–2217. 3044995 10.1128/iai.56.9.2209-2217.1988 PMC259551

[31] Stanley RA, Ram SP, Wilkinson RK, Roberton AM (1986) Degradation of pig gastric and colonic mucins by bacteria isolated from the pig colon. Appl Environ Microbiol 51: 1104–1109. 2873788 10.1128/aem.51.5.1104-1109.1986 PMC239018

[32] Roberton AM, Stanley RA (1982) In vitro utilization of mucin by Bacteroides fragilis. Appl Environ Microbiol 43: 325–330. 6174077 10.1128/aem.43.2.325-330.1982 PMC241826

[33] Prizont R, Reed WP (1991) Differences in blood group B-specific mucinase activity between virulent and avirulent Shigella flexneri 2a strains. Microb Pathog 11: 129–135. 1961109 10.1016/0882-4010(91)90006-v

[34] Pastorello I, Rossi Paccani S, Rosini R, Mattera R, Ferrer Navarro M, et al (2013) EsiB, a novel pathogenic Escherichia coli secretory immunoglobulin A-binding protein impairing neutrophil activation. MBio 4. 10.1128/mBio.00206-13 PMC3735183 23882011

[35] Zisakis A, Katsetos Cd, Vasiliou D, Karachalios T, Sakkas L (2007) Expression of Retinoic Acid Receptor (RAR) alpha Protein in the Synovial Membrane from Patients with Osteoarthritis and Rheumatoid Arthritis. Int J Biomed Sci 3: 46–49. 23675020 PMC3614619

[36] Vieira MA, Gomes TA, Ferreira AJ, Knobl T, Servin AL, et al (2010) Two atypical enteropathogenic Escherichia coli strains induce the production of secreted and membrane-bound mucins to benefit their own growth at the apical surface of human mucin-secreting intestinal HT29-MTX cells. Infect Immun 78: 927–938. 10.1128/IAI.01115-09 20065027 PMC2825950

[37] Gouyer V, Wiede A, Buisine MP, Dekeyser S, Moreau O, et al (2001) Specific secretion of gel-forming mucins and TFF peptides in HT-29 cells of mucin-secreting phenotype. Biochim Biophys Acta 1539: 71–84. 11389969 10.1016/s0167-4889(01)00092-1

[38] Dolan B, Naughton J, Tegtmeyer N, May FE, Clyne M (2012) The interaction of Helicobacter pylori with the adherent mucus gel layer secreted by polarized HT29-MTX-E12 cells. PLoS One 7: e47300 10.1371/journal.pone.0047300 23056622 PMC3466223

