# Supplementary figures and images for: Pathogenic E. coli Exploits SslE Mucinase Activity to Translocate through the Mucosal Barrier and Get Access to Host Cells
Source: PLoS One. 2015 Mar 19;10(3):e0117486. doi: 10.1371/journal.pone.0117486 (PMC4366376; doi:10.1371/journal.pone.0117486)

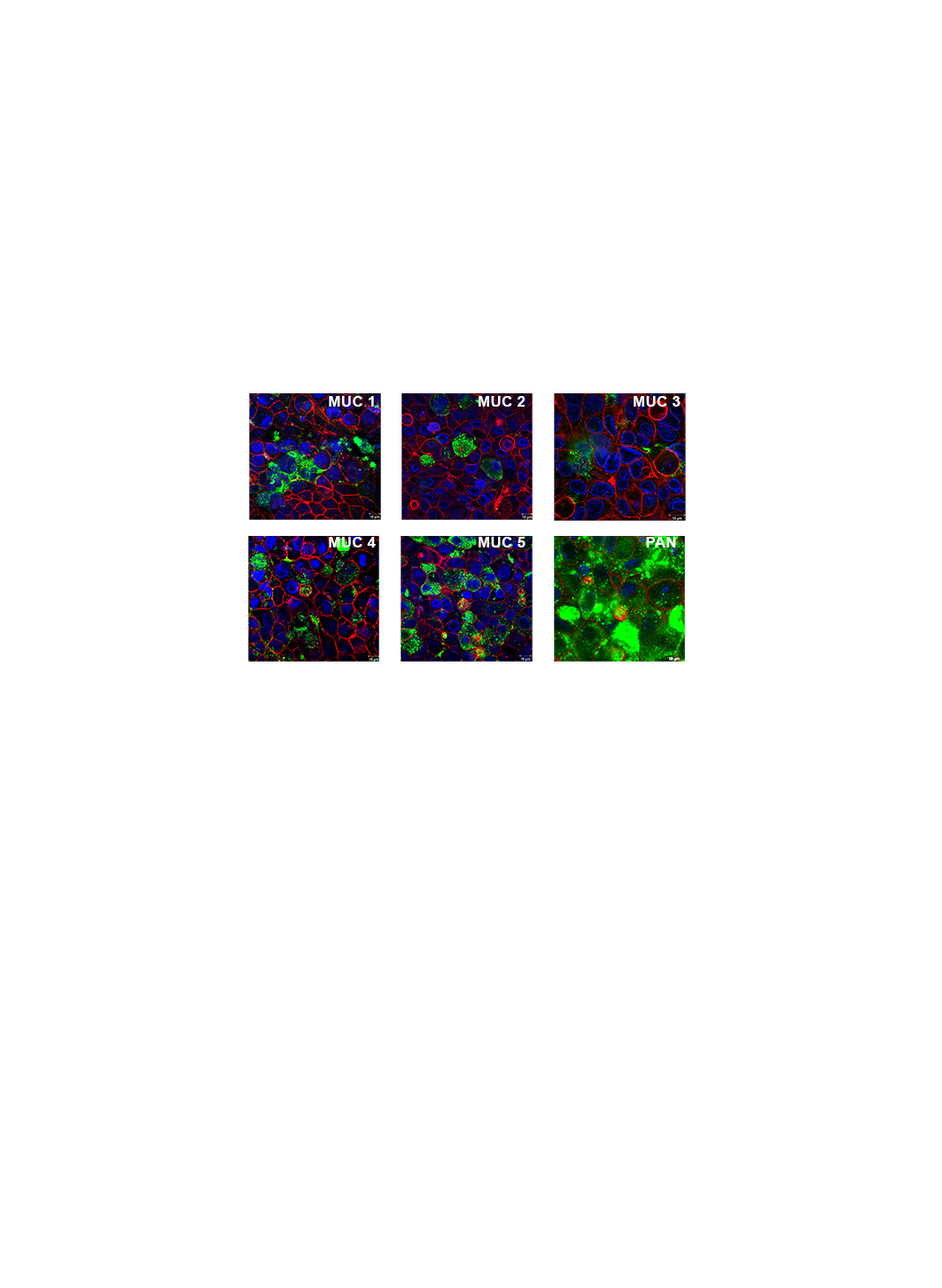

Supplement: S1 Fig — HT29-MTX cells grown on transwell filters for 21 days were stained with specific antibodies for MUC1, MUC2, MUC3, MUC4,MUC5AC and PAN anti-gastric mucin. The mucins are stained in green and the actin skeleton in red. DAPI (blue) staining was used to visualize cell nuclei. (TIF) [file pone.0117486.s001.tif]

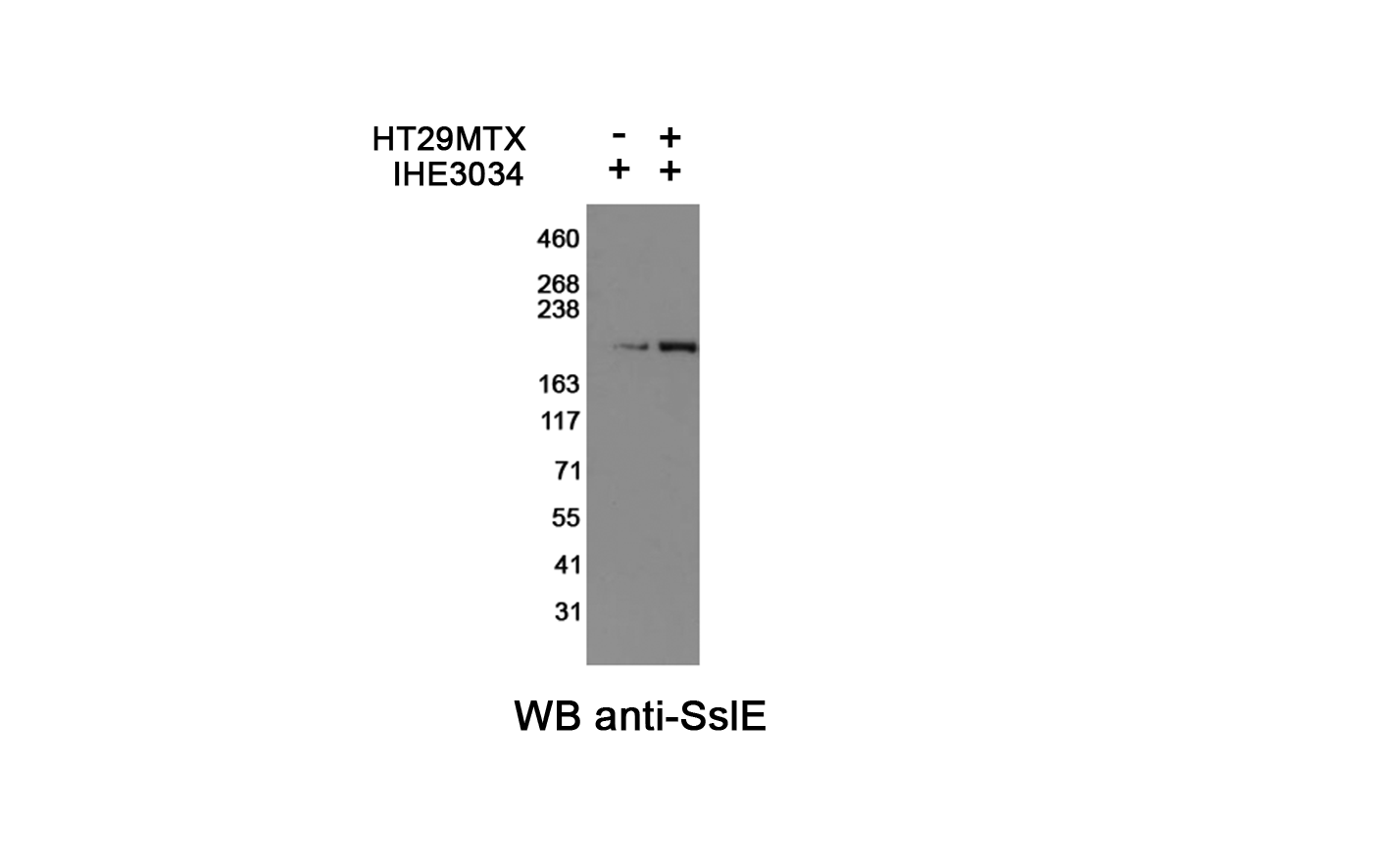

Supplement: S2 Fig — Immunoblot analysis of SslE expression in S/N of bacteria incubated with medium alone (control) or differentiated HT29-MTX cells. A representative experiment out of 3 performed is shown. Molecular weight markers are indicated on the left column. (TIF) [file pone.0117486.s002.tif]
